# High-Frame-Rate Echocardiography: A New Frontier in Noninvasive Functional Assessment

**DOI:** 10.3390/jcm15062460

**Published:** 2026-03-23

**Authors:** Fatemeh Mashayekhi, Fatemeh Shahbazi, Andressa Araujo Andrade Sousa, Miaomiao Liu, Jens-Uwe Voigt, Annette Caenen, Jan D’hooge

**Affiliations:** 1 Department of Cardiovascular Sciences, Katholieke Universiteit Leuven (KU Leuven), 3000 Leuven, Belgium; fatemeh.mashayekhi@kuleuven.be (F.M.); fatemeh.shahbazi@kuleuven.be (F.S.); andressa.araujo@kuleuven.be (A.A.A.S.); miaomiao.liu@kuleuven.be (M.L.); jens-uwe.voigt@kuleuven.be (J.-U.V.); annette.caenen@kuleuven.be (A.C.); 2 Department of Cardiovascular Diseases, University Hospital Leuven, 3000 Leuven, Belgium; 3 Department of Electronics and Information systems, Ghent University, 9000 Ghent, Belgium

**Keywords:** high frame rate, ultrafast, echocardiography, deformation imaging, mechanical wave imaging, shear wave imaging, shear wave elastography, flow imaging

## Abstract

High-frame-rate (HFR) ultrasound imaging enables the acquisition of up to several thousand frames per second, substantially improving the temporal resolution of echocardiography. This technical advancement allows visualization of rapid mechanical and hemodynamic events that are not captured by conventional systems. In this review, we summarize the methods used to achieve HFR acquisition and examine their application across three principal domains: deformation imaging, mechanical wave imaging, and blood flow imaging. In deformation imaging, clinical studies have demonstrated higher feasibility for myocardial motion tracking and more reliable temporal deformation parameters. Mechanical wave imaging has emerged as a complementary domain, using HFR acquisition to capture transient mechanical events and estimate regional myocardial stiffness under both physiological and pathological conditions. In flow imaging, improved temporal resolution enables detailed visualization of rapid intracardiac flow and the evaluation of complex hemodynamic patterns. This technology expands the scope of functional and quantitative cardiac assessment and is emerging as a valuable modality for noninvasive diagnosis and monitoring in cardiovascular disorders.

## 1. Introduction

Echocardiography has evolved from its experimental origins in the early 1950s into an indispensable standard clinical imaging modality that integrates structural, functional, and flow assessment. However, conventional systems acquire only 40–90 frames per second, limiting their ability to capture fast and short-lived cardiac events [1]. Recent technological progress has enabled high-frame-rate (HFR) ultrasound, which overcomes this limitation by acquiring thousands of frames per second [1,2,3,4,5,6]. In deformation imaging, high frame rate improves the robustness of strain and motion tracking during rapid cardiac phases [7,8,9,10,11]. In mechanical wave imaging, it enables capturing mechanical waves of minute amplitude propagating at high speed through the myocardium, allowing estimation of myocardial stiffness [12]. In contrast to flow imaging, it enables visualization of fast and complex hemodynamics, allowing quantification of intracardiac velocities and vortical flow patterns [13,14].

**Figure 1:**
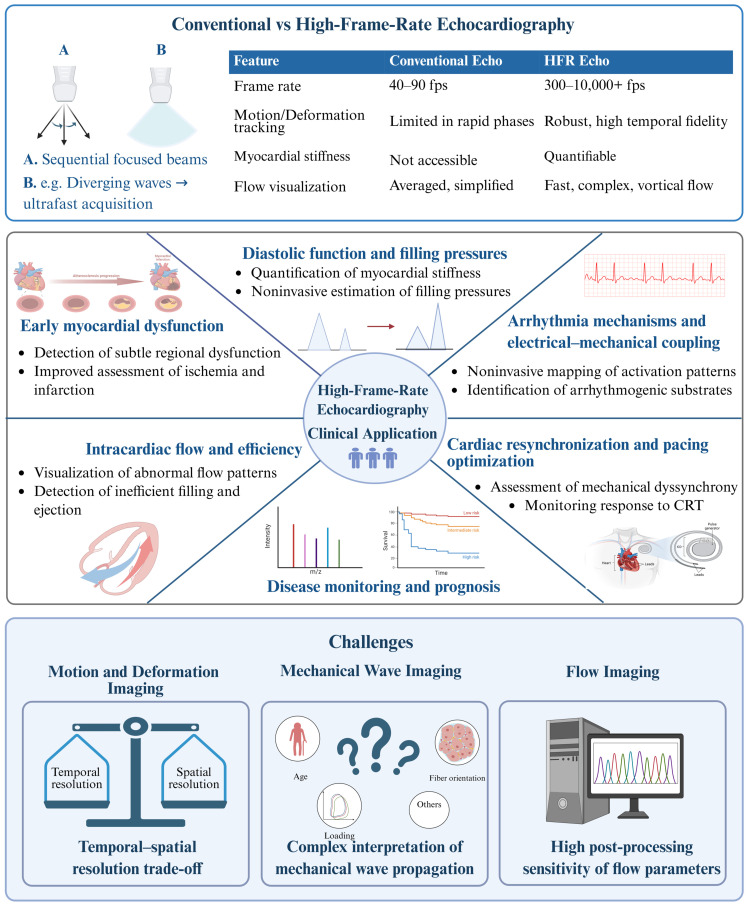
Central illustration highlighting the advantages of high-frame-rate echocardiography over conventional imaging, its main clinical applications, and key technical challenges.

In recognition of the potential of HFR imaging, the ASE guidelines on left ventricular diastolic function and heart failure with preserved ejection fraction diagnosis mentioned shear wave elastography as a promising research application and emphasized the need for multicenter validation [15]. The field now stands on the verge of broader clinical adoption. This review outlines the technical foundations and clinical applications of HFR echocardiography, highlighting its strengths, limitations, and future perspectives. It first describes how high frame rates are achieved, then examines deformation, mechanical wave, and flow imaging through their principles and clinical use. Figure 1 summarizes the main domains discussed in the paper.

## 2. How to Achieve High Frame Rates?

**Figure 2:**
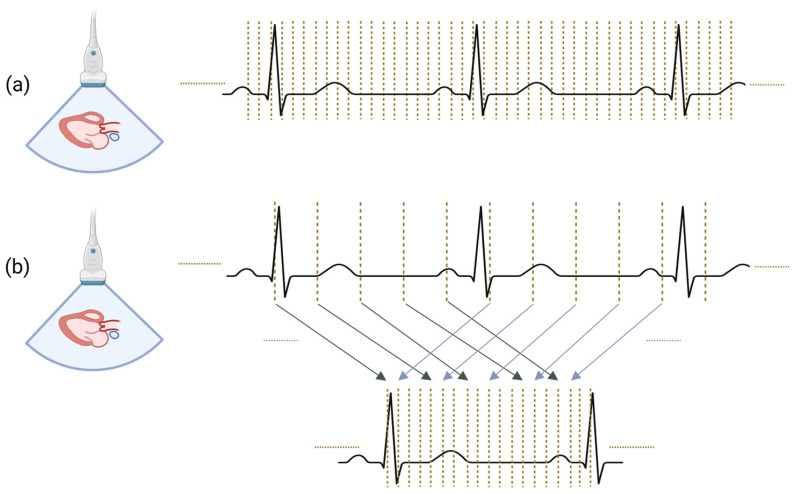
Conceptual illustration of strategies used to achieve high-frame-rate ultrasound imaging. Dashed lines represent imaging events. (**a**) Direct approaches: more frames are acquired within a given time interval by modifying the transmit–receive sequence. (**b**) Indirect approaches: the effective frame rate is increased through post-processing of previously acquired frames. In this schematic, sparsely sampled frames from successive cardiac cycles are retrospectively reordered and combined to reconstruct a sequence with higher effective temporal resolution. Arrows indicate imaging events acquired in different cardiac cycles that are reused and reassigned to new temporal positions in the reconstructed sequence.

HFR ultrasound can be implemented through two main strategies: direct approaches, which modify the transmission and acquisition process, and indirect approaches, which enhance frame rate through post-processing. This distinction is illustrated in Figure 2, where dashed lines indicate individual imaging events.

### 2.1. Indirect Approaches

Indirect approaches enhance temporal resolution by increasing the sampling rate or by up-sampling, without altering the transmit–receive sequence. The following methods increase the effective sampling rate by combining information across acquisitions. Retrospective ECG gating has been used to reconstruct high-temporal-resolution image sequences by combining data from different cardiac cycles [16]. Sliding window compounding improves temporal resolution by averaging overlapping sets of consecutive frames, allowing partial reuse of transmit data across neighboring acquisitions [17,18]. In contrast, the following methods improve temporal continuity retrospectively, as they operate on previously acquired data rather than generating new temporal samples. Frame interpolation synthetically generates intermediate frames between acquisitions to increase the perceived frame rate [19,20,21,22,23]. Additional post-processing strategies, including frame reordering [24], manifold learning [25], and sparse representation techniques [26], have also been developed to enhance temporal resolution after acquisition.

### 2.2. Direct Approaches

Direct approaches overcome the intrinsic frame-rate limitation of conventional single-line transmission, where only one focused line is acquired per transmit. Frame rate is primarily determined by the sound propagation speed, imaging depth, and number of transmit events used to reconstruct an image. Since sound velocity and imaging depth are fixed by physical and anatomical constraints, respectively, increasing frame rate requires reducing the number of transmit events per image. Several strategies address this: in multi-line transmit, multiple focused beams are emitted simultaneously [27], whereas in multi-line acquisition, several receive lines are reconstructed from each transmit event [28]. Plane-wave imaging transmits a single unfocused broad beam that insonifies the entire field of view, i.e., equal to the width of the transducer aperture [12], while diverging-wave imaging emits a spherical wavefront that expands outward from the probe to cover a wide region [29]. In both cases, all transducer elements receive echoes originating from the entire field of view. Data from all elements is used for real-time retrospective reconstruction of synthetic scan lines by applying appropriate receive delays. Although broad transmit beams degrade image quality in terms of resolution and contrast, coherent compounding can mitigate this by transmitting several wide beams at slightly different angles and combining them into a single image. However, this enhancement comes at the cost of temporal resolution due to the additional transmits required [12].

The following sections are organized according to the main functional domains of HFR echocardiography. For each modality, the technical principles are first outlined, followed by representative experimental and clinical studies that demonstrate their diagnostic value.

## 3. Motion and Deformation Assessment at High Frame Rate

A detailed overview of the clinical studies on motion and deformation imaging discussed in this paper is provided in Table S1 (Supplementary Materials).

### 3.1. Principles and Techniques of High-Frame-Rate Motion and Deformation Imaging

Deformation imaging quantifies how myocardial segments shorten and lengthen, providing a regional assessment of myocardial performance beyond global indices such as ejection fraction. The two principal parameters are strain, the relative change in myocardial length with respect to its resting dimension, and strain rate, the temporal derivative of strain describing the speed of deformation [30].

Strain and strain-rate imaging can be derived from tissue Doppler imaging (TDI) or speckle-tracking echocardiography (STE). TDI measures velocities based on color Doppler algorithms (i.e., auto-correlation of multiple pulses along a scanline); consequently, velocity quantification is reliable only when motion is aligned with the direction of insonation [31]. In contrast, STE is a post-processing method that tracks the displacement of speckles, natural acoustic markers, across consecutive B-mode frames. A block-matching algorithm is used to estimate speckle motion in any imaging plane, allowing strain to be derived in any desired direction within a defined region of interest [32]. It is noteworthy that speckle tracking across the ultrasound beam is less accurate than tracking along the beam, particularly in the far field. This reduced accuracy reflects the directional resolution characteristics of sector imaging, as demonstrated by Langeland et al. [33]. Owing to its reproducibility and ease of implementation, STE has become the preferred clinical approach.

In conventional echocardiography, tissue Doppler imaging provides relatively high temporal resolution (about 100 fps for a full sector and up to 400 fps for a narrow sector) because only a small number of Doppler beams are used to cover the sector. However, the temporal resolution of speckle tracking is constrained by the frame rate of the underlying B-mode images, which is typically around 60 fps [32,34,35]. Both tissue Doppler and speckle tracking can be applied to high-frame-rate imaging data, providing the temporal sampling necessary to characterize rapid cardiac motion and deformation more accurately.

Hybrid approaches have been proposed to integrate the higher temporal resolution of Doppler-based techniques with the multidirectional tracking capability of STE. Blessberger and Binder [36] emphasized that combining information from both modalities enhances sensitivity to regional deformation and enables a more comprehensive assessment of myocardial function. More recent developments, such as motion-compensated high-frame-rate STE, further improved accuracy by correcting for tissue motion during coherent compounding and by incorporating optical-flow-based subpixel refinement, allowing reliable estimation of two-dimensional myocardial velocities and strain at several hundred frames per second [37].

### 3.2. Experimental and Clinical Applications of High-Frame-Rate Motion and Deformation Imaging

In the following subsections, studies are organized by their intended objective, with clinical applications discussed within each group.

#### 3.2.1. Imaging Myocardial Mechanics

Early work of Slørdahl et al. [38] demonstrated the feasibility of strain-rate imaging in the interventricular septum of healthy subjects, revealing distinct mechanical events during isovolumic contraction and relaxation. This study established the ability of deformation imaging to resolve rapid changes in myocardial mechanics and provided the basis for subsequent developments in strain-based imaging.

In stress echocardiography, Orlowska et al. [11] reported reproducible strain and strain-rate responses across views and loading conditions using HFR sequences, supporting feasibility during elevated heart rates. For diastolic evaluation, Papangelopoulou et al. [39] showed that HFR speckle tracking aligns more closely with Doppler indices during isovolumic relaxation and early filling than conventional acquisitions, strengthening clinical confidence in diastolic markers. Porée et al. [40] combined Doppler information with optical-flow tracking to improve multidirectional velocity estimation, which translates clinically into more consistent regional strain interpretation when motion patterns are complex.

Clinically, Schleifer et al. [41] demonstrated that strain imaging at the point of care differentiates myocardial infarction from angina in emergency presentations, revealing markedly reduced principal strain in infarcted myocardium. El Harake et al. [42] extended this concept to stress testing, introducing the strain-difference metric (Δε) to quantify ischemia-related impairment and validate detection against nuclear perfusion imaging.

#### 3.2.2. Imaging Surrogates of Electrical Activation

**Figure 3:**
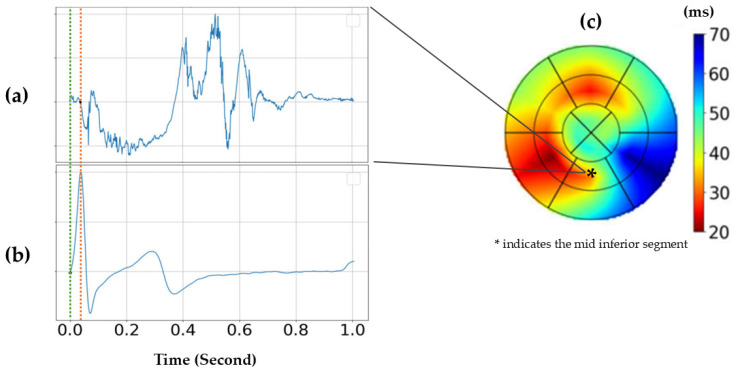
Representative example illustrating high-frame-rate mechanical activation analysis in a healthy volunteer: (**a**) scaled segmental left ventricular strain-rate curve from the mid inferior segment (segment 10); (**b**) corresponding ECG; and (**c**) left ventricular mechanical activation map, with the mid inferior segment highlighted (*). The color scale represents the time delay between the onset of QRS (green line) and the first significant negative deflection of the strain rate curve of the respective myocardial segment (red line). Adapted from Safarzadeh et al. [46].

High-frame-rate acquisition enables advanced mapping of the electromechanical activation sequence. Electromechanical wave imaging (EWI) visualizes the propagation of contraction following electrical depolarization, allowing noninvasive estimation of local activation timing as the interval between QRS onset and the first zero-crossing of the strain-rate curve [39,43,44,45]. This approach provides insight into the regional pattern of the onset of myocardial shortening and synchrony. Such activation maps can be generated automatically from HFR strain-rate data using patient-specific spatiotemporal analysis, as demonstrated by Safarzadeh et al. [46], from whom Figure 3 is adapted.

Provost et al. [43] first demonstrated the clinical feasibility of EWI by mapping atrial electromechanical coupling in patients with arrhythmias, including premature ventricular complexes, focal tachycardia, atrial flutter, and atrial fibrillation. Spatiotemporal strain maps delineated both focal and reentrant activation pathways, showing strong agreement between mechanical and electrical activation cycles. This work established EWI as a promising tool for arrhythmia characterization and ablation planning. Subsequent studies refined both spatial resolution and diagnostic accuracy [44,45]. Bessière et al. [44] demonstrated in isolated swine hearts that EWI can differentiate endocardial from epicardial activation within the ventricular wall, whereas Tonko et al. [45] confirmed in patients with ventricular tachycardia that EWI accurately localizes arrhythmia origins, consistent with invasive electroanatomic mapping.

Tourni et al. [47] used EWI to assess electromechanical activation of the mitral valve in pediatric subjects with prolapse and regurgitation, revealing delayed papillary-muscle activation consistent with impaired valve–ventricular coupling and potential arrhythmogenic substrate. Melki et al. [48] introduced an EWI-derived dispersion metric to quantify ventricular activation synchrony across pacing strategies, showing that His-bundle pacing achieved the most physiological activation pattern with the lowest dispersion compared with biventricular and right-ventricular pacing.

EWI extensions, such as electromechanical cycle-length mapping (ECLM), analyze the frequency content of strain or strain-rate signals to determine the dominant electromechanical cycle length at each myocardial location [49]. This spectral approach enables mapping of local activation periodicity over multiple cardiac cycles. Tourni et al. [49,50,51] used ECLM to assess atrial activation and guide therapy in atrial fibrillation. In patients undergoing cardioversion, ECLM identified regions associated with early recurrence and differentiated those who maintained sinus rhythm from those who relapsed [49]. Subsequent studies [50,51] extended its use to ablation, showing that pre-procedural electromechanical dispersion predicted long-term recurrence and that post-procedural mapping could confirm effective elimination of arrhythmic triggers.

Deformation imaging techniques, encompassing strain, strain-rate, and electromechanical wave imaging, have shown wide-ranging diagnostic and interventional potential. Applications now extend from noninvasive mapping of arrhythmic activity and risk stratification to the assessment of ischemia, mechanical dyssynchrony, and valvular dysfunction.

These studies highlight that adequate temporal resolution is required to capture higher-frequency components of myocardial deformation during short cardiac phases, such as isovolumic contraction and relaxation, which are often undersampled at conventional frame rates. By improving temporal sampling, HFR deformation imaging allows more accurate timing of regional mechanical activity and enables noninvasive mapping of myocardial activation sequences with electromechanical wave imaging, which may support arrhythmia localization and pacing assessment [43,44,45,46,47,48].

## 4. Imaging Mechanical Waves to Assess Myocardial Properties

While ultrasound imaging relies on longitudinal waves for image formation, additional mechanical waves can propagate within the myocardium and can be analyzed for tissue stiffness assessment; these are referred to as mechanical waves in this paper. The clinical studies reviewed in this paper that implement mechanical waves are summarized in Table S2 (Supplementary Materials).

### 4.1. Principles and Techniques of Mechanical Wave Imaging

**Figure 4:**
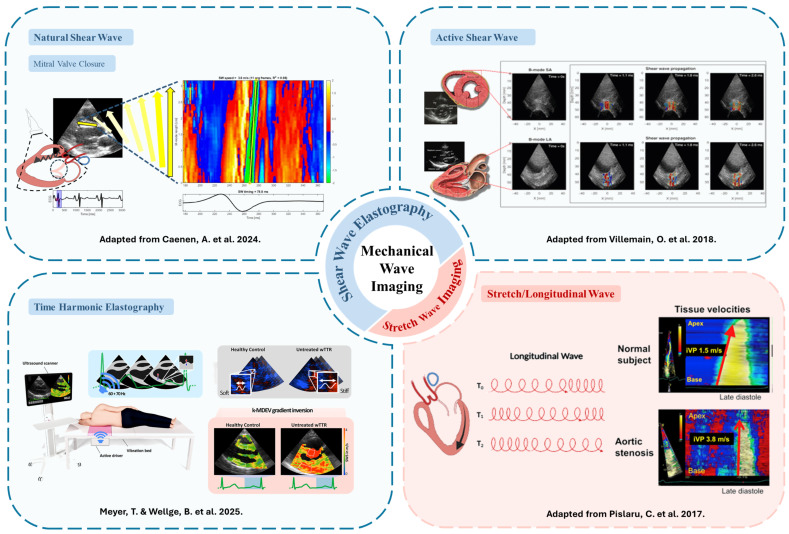
Overview of principal modalities used in mechanical wave propagation imaging for myocardial stiffness assessment. (**Top left**): natural shear waves generated after mitral valve closure. (**Top right**): active shear waves induced by focused ultrasound excitation. (**Bottom left**): time harmonic elastography using external vibration for stiffness mapping. (**Bottom right**): stretch waves or longitudinal waves propagating from base to apex during ventricular filling. Adapted from Caenen et al. [62], Villemain et al. [66], Meyer and Wellge et al. [69], and Pislaru et al. [57], respectively.

Observing the propagation of mechanical waves through myocardium provides a noninvasive approach to quantify myocardial stiffness (MS) as waves travel faster in stiff media. Two main types of mechanical waves are investigated: shear waves (SW), in which particle displacement occurs transversely to the propagation direction, whose speed reflects tissue stiffness [52,53,54,55,56], and longitudinal stretch waves, also referred to as intrinsic velocity propagation (iVP) or atrial kick waves, which travel from base to apex during ventricular filling and are related to myocardial elasticity [57,58]. Both types of waves depend on the mechanical properties of the myocardium but arise from different physical principles. Shear waves follow the shear-wave equation obtained from the Navier–Cauchy equations of linear elasticity, linking propagation speed to the shear modulus, whereas stretch waves are described by the Moens–Korteweg model, which relates wave velocity to Young’s modulus, wall curvature (radius), and density. Figure 4 summarizes the main mechanical wave imaging modalities reviewed in this paper.

Mechanical wave imaging can be implemented using different excitation mechanisms, including natural cardiac events, active probe-based excitation, and harmonic excitation via external actuators, as well as different tracking strategies such as tissue Doppler and clutter filtering. Malik et al. [59] compared active and natural shear wave approaches in patients, highlighting differences related to excitation mechanism and cardiac phase. Each approach relies on distinct mechanisms for wave generation and propagation assessment, and offers specific technical and clinical advantages and challenges.

Natural shear waves and stretch waves originate from physiological events such as mitral and aortic valve closure [60,61,62], which generate transient mechanical impulses causing a vibration that propagates through the myocardium. These waves induce relatively large tissue displacements, typically on the micrometer scale, with relatively high signal-to-noise ratios, allowing tracking over longer distances; however, they occur only at two distinct time points of the cardiac cycle.

In active shear wave elastography (SWE), a focused ultrasound push pulse (duration < 1 ms) is used to generate localized displacements via acoustic radiation force (ARF), offering precise control over the place and time of excitation, allowing targeted assessment of MS [63,64]. Depending on when the shear waves are excited during the cardiac cycle, their measurements can provide access to parameters such as contractility [64], relaxation properties [65], passive myocardial stiffness [66], or myocardial work [67].

For both, active and natural SWE, wave propagation is commonly detected using tissue Doppler imaging, and their propagation is traced in an M-mode-like representation of the myocardial wall. Manual or automated algorithms are then used to estimate the propagation speed of the wave front (Figure 4). For this, the imaging direction needs to be roughly perpendicular to the propagation direction of the wave in the myocardium.

Clutter-filter-based approaches have also been explored [68], in which temporal high-pass filtering is applied to beamformed ultrasound data to suppress slow background tissue motion and enhance the faster components associated with propagating mechanical waves. The filtered images display the propagating mechanical events as dark wavefronts across the myocardium, enabling their visualization without explicit motion tracking.

Echocardiographic harmonic elastography, introduced by Meyer et al. [69], has been adopted from CMR elastography and applies continuous low-frequency external vibrations beneath the thorax to generate harmonic waves that propagate through the myocardium. Using ECG-gated imaging (see Section 2.1), tissue Doppler-derived displacement fields are reconstructed into harmonic motion maps, providing an indirect, high-temporal-resolution evaluation of myocardial stiffness.

A comprehensive overview of shear-wave generation mechanisms, acquisition strategies, and processing methods can be found in recent reviews by Caenen et al. [62] and Wouters et al. [70].

### 4.2. Experimental and Clinical Applications of Mechanical Wave Propagation Imaging

#### 4.2.1. Experimental and Clinical Applications of Shear Wave Elastography

The first measurement of natural shear wave speed (SWS) following aortic valve closure was reported by Kanai in 2005 [71], and subsequent studies have established reference values in healthy populations [72]. Villemain et al. [66] and Malik et al. [73] demonstrated a linear relation between myocardial stiffness, measured with active SWE, and age in healthy adults and children. Using natural shear waves, Youssef et al. [74] also reported an age-related increase in shear wave speed, with a particularly pronounced rise during the first months of life. The authors hypothesized that this behavior reflects concurrent changes in ventricular geometry, such as increasing wall thickness, and in myocardial composition, including collagen content.

##### Cardiomyopathies

Across multiple studies, SWE has consistently shown elevated speed in cardiomyopathies compared to healthy subjects. In an early study, Villemain et al. [54,66] applied active SWE in both pediatric and adult hypertrophic cardiomyopathy, demonstrating significantly increased end-diastolic myocardial stiffness compared with healthy controls, with particularly elevated values in pediatric patients exhibiting restrictive physiology. Both studies also introduced a shear-wave-based fractional anisotropy metric, which was reduced in HCM, consistent with altered myocardial microstructural organization [54,66]. In parallel, Strachinaru et al. [75] reported increased shear wave velocities in adult HCM using naturally occurring shear waves generated by mitral and aortic valve closure.

Petrescu et al. [76] reported higher natural SWS after both mitral and aortic valve closure in patients with cardiac amyloidosis (CA), with strong correlations to E/e′ and diastolic dysfunction grade. Cafezeiro et al. [77] also found increased stiffness in CA and Fabry disease compared with controls, though some overlap limited differentiation between phenotypes. Using externally induced harmonic waves, Meyer et al. [69] observed significant differences in stiffness between CA, moderate left ventricular hypertrophy, and control groups, as well as a post-tafamidis reduction in stiffness, supporting SWE’s potential for treatment monitoring.

##### Valve Diseases

Natural shear waves are especially sensitive to structural changes in the valves. Wouters et al. [78] examined valve-related wave morphology in patients with native aortic stenosis, post-valve replacement, and healthy controls, observing reduced amplitude in aortic stenosis cases and shorter temporal width post-TAVI.

Myocardial changes, induced by valve disease, may also be detected by SWE. Espeland et al. [79] evaluated shear-wave propagation following aortic valve closure and atrial contraction in patients with aortic stenosis and healthy volunteers. Wave velocities were significantly higher after aortic valve closure in the patient group, while atrial contraction-related waves showed no difference.

##### Myocardial Fibrosis

SWE has been applied to assess myocardial fibrosis across a range of structural and infiltrative cardiac conditions. Wouters et al. [80] employed natural SWE to detect septal scarring in CRT candidates with left bundle branch block (LBBB), finding higher septal SWS in infarcted than in non-infarcted patients. Speeds also increased when biventricular pacing was deactivated, indicating mechanical dyssynchrony.

In heart transplant recipients, Petrescu et al. [81] measured naturally induced shear waves after mitral valve closure and found strong correlations with CMR-derived T1 values as an indicator of diffuse myocardial fibrosis. The correlation was stronger than that of T1 values and pulmonary capillary wedge pressure, indicating that SWE might be a sensitive modality for the follow-up of HTx patients, sparing repetitive invasive measurements.

In hypertensive heart disease, SWE has been used to differentiate concentric remodeling from concentric hypertrophy. Cvijic et al. [82] measured SWS at mitral valve closure and found near-normal values in concentric remodeling but marked elevation in concentric hypertrophy, a disease state where myocardial fibrosis sets in. SWS correlated with structural and functional indices of remodeling and remained elevated after wall-stress normalization, indicating that SWE primarily reflects intrinsic myocardial changes over passive loading.

##### Diastolic Dysfunction

As stiffness is a main determinant of diastolic function, SWE has been explored as a tool for assessing ventricular filling dynamics. Werner et al. [83] investigated natural SWE as a quantitative marker of diastolic function, showing a strong correlation between shear wave speed (SWS) after mitral valve closure and invasively measured left ventricular filling pressures, with SWE outperforming guideline-based echocardiographic indices. In the same paper, a study following acutely decompensated heart failure patients during recovery showed a significant decrease in SWS after diuretic therapy, supporting the relation of SWS and filling pressures. Similarly, Bézy et al. [84] investigated the effect of preload reduction during hemodialysis and confirmed that SWS decreases with fluid removal, particularly in heart failure patients, reflecting load dependence while retaining sensitivity to intrinsic myocardial stiffness. Salaets et al. [85] extended these findings to children with a Fontan circulation, showing increased diastolic stiffness and steeper age-related progression, emphasizing SWE’s value for characterizing ventricular stiffness also in more complex circulations. Youssef et al. [86] demonstrated elevated myocardial stiffness in multisystem inflammatory syndrome following COVID-19 despite preserved systolic function.

##### Right Ventricular Applications

Right ventricular (RV) dysfunction is an important prognostic factor across cardiovascular diseases [87,88,89,90]. Compared with the left ventricle, where SWE has been applied in various conditions, RV studies remain limited and preliminary, partly due to the less favorable anatomical position and accessibility of the RV walls. Most available clinical data come from pediatric pressure-overload states: Venet et al. [91] performed the first in vivo assessment of RV stiffness using natural SWE in children with pulmonary hypertension, showing that SWS increased with RV pressure load. Adult evidence is scarce, but small series in transthyretin cardiac amyloidosis (ATTR-CM) have reported elevated RV stiffness correlating with remodeling indices and biomarkers, and inversely with functional measures [77,92].

These studies suggest that SWE can detect RV stiffening and may support a quantitative assessment of right ventricular mechanics, but evidence remains limited.

#### 4.2.2. Experimental and Clinical Applications of Stretch Wave Imaging

Stretch wave imaging was first demonstrated in early echocardiographic studies, identifying a distinct diastolic wave during ventricular relaxation. Støylen et al. [93] showed that the velocity of this wave decreases in impaired relaxation, linking it to diastolic dysfunction. Building on these early observations, Pislaru et al. [57] applied the technique clinically in patients with severe aortic stenosis and mitral regurgitation, reporting elevated propagation velocities in aortic stenosis and intermediate values in mitral regurgitation. Velocities correlated with septal E/e′ and global longitudinal strain, enabling early identification of abnormal compliance, whereas color M-mode propagation showed limited discriminatory value.

The technique was later applied to patients with amyloidosis. Pislaru et al. [94] demonstrated markedly elevated intrinsic velocity propagation (iVP) in CA compared with non-cardiac amyloidosis and healthy controls, with strong correlations to wall thickness, chamber stiffness indices, biomarkers, and global longitudinal strain. A clinically defined iVP threshold distinguished patients at higher risk of adverse events, independent of conventional echocardiographic parameters [94,95]. In a recent multimodality study, Benz et al. [96] applied stretch wave imaging to assess myocardial stiffness in healthy subjects and in patients with CA and HCM. iVP correlated strongly with extracellular volume and amyloid burden on cardiac MRI, confirming its accuracy for noninvasive stiffness evaluation and its diagnostic relevance in amyloidosis.

Strachinaru et al. [97] evaluated myocardial stretch wave after atrial contraction in healthy volunteers and patients with hypertrophic cardiomyopathy, showing similar average propagation velocities between groups but non-constant propagation patterns in a subset of patients. This behavior was associated with septal thickness and was not correlated with conventional diastolic indices.

Using three-dimensional high-frame-rate echocardiography, Halvorsrød et al. [98] reported higher iVP in infarcted regions compared with remote myocardium and healthy controls in acute myocardial infarction. Salles et al. [99] demonstrated the feasibility of clutter-filter wave imaging with three-dimensional high-frame-rate echocardiography, observing higher wave velocities in infarcted segments than in remote regions.

Andresen et al. [100] assessed iVP in acute coronary syndrome, finding greater feasibility for stretch waves than for natural waves (mitral and aortic valve closure). Elevated iVP was independently associated with wall motion abnormalities and regional dysfunction. In general, stretch wave imaging appears to be less feasible in parasternal images.

Taken together, these studies demonstrate that HFR imaging enables visualization and tracking of fast mechanical waves propagating through the myocardium, which cannot be captured at conventional echocardiographic frame rates, offering a noninvasive approach to assess myocardial stiffness. Clinically, shear wave and stretch wave imaging have demonstrated the ability to detect increased myocardial stiffness in cardiomyopathies such as hypertrophic cardiomyopathy and cardiac amyloidosis and to characterize structural remodeling, including myocardial fibrosis and elevated ventricular filling pressures.

## 5. Flow Imaging

A summary of the clinical studies on HFR flow imaging cited in this paper is presented in Table S3 (Supplementary Materials).

### 5.1. Principles and Techniques of Flow Imaging

Cardiac function is governed not only by myocardial contractility and valvular integrity, but also by the efficiency of intracardiac blood flow. Hemodynamic abnormalities may appear not only in congenital abnormalities but also early in functional disease, even when structural changes are minimal. HFR ultrasound enables capturing intracardiac flow either without contrast, by tracking native blood speckles, or with contrast agents, by tracking microbubbles.

Blood speckle tracking (BST) directly visualizes and quantifies intracardiac flow by tracking the natural speckle pattern produced by red blood cells. Echo particle image velocimetry (echoPIV) quantifies intracardiac flow by tracking ultrasound contrast agent microbubbles, adapting the principles of optical particle image velocimetry to echocardiography [101]. In practice, small kernels of the speckles are identified in one frame, and their displacement is detected in the following frame, providing both the magnitude and direction of local blood motion, as described by Nyrnes et al. [102]. In both cases, HFR imaging provides the temporal resolution required to capture rapid flow phenomena.

From velocity vector fields, additional hemodynamic parameters can be derived through post-processing. Vorticity, defined as the local curl of the velocity field, describes the rotational component of blood motion and enables identification of intraventricular vortices as coherent regions of circulating flow [101,103]. The role of these vortices in organizing ventricular filling and facilitating efficient flow redirection has been described in experimental and computational fluid dynamic studies [104,105]. These flow features have been used to characterize intraventricular flow organization during diastolic filling. Using the same velocity gradients, intracardiac energy loss can be estimated by quantifying viscous dissipation, providing a measure of energetic inefficiency associated with disturbed diastolic flow patterns [102].

### 5.2. Experimental and Clinical Applications of Flow Imaging

Although modern commercial ultrasound platforms now allow for clinical blood flow assessment [106], translation of high-frame-rate intracardiac flow imaging into routine practice remains largely exploratory, as its added diagnostic value continues to be investigated. The following sections summarize efforts to advance HFR flow imaging toward clinical implementation.

Fadnes et al. [107] used a high-frame-rate 2D blood speckle tracking technique to assess shunt flow in neonates with atrial and ventricular septal defects. The method enabled angle-independent visualization of shunt dynamics and provided higher peak velocity estimates than Doppler, capturing bidirectional and time-varying flow. Nyrnes et al. [102] confirmed the feasibility of BST across a broad pediatric cohort from fetuses to 11-year-olds, showing reliable visualization of intracardiac flow phenomena across age groups. Cantinotti et al. [108] demonstrated the utility of BST in neonates with complex congenital defects, including transposition of the great arteries, tetralogy of Fallot, and aortic coarctation. BST visualized shunt direction, vortices, regurgitant jets, and stenotic flow propagation with great detail and, in some cases, greater clarity than conventional color Doppler, even in unsedated neonates. In a large pediatric cohort, Sørensen et al. [8] applied high-frame-rate BST to quantify early-diastolic intraventricular pressure difference (IVPD), reporting reduced values in dilated and hypertrophic cardiomyopathies. In a separate prospective study, the same group described physiological maturation of diastolic flow in infants: left ventricular energy loss, kinetic energy, and vorticity changed during the first weeks of life, while IVPD continued to increase up to six months [109].

**Figure 5:**
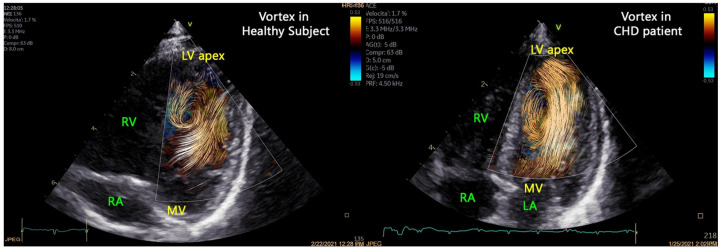
Adapted illustration of left ventricular vortices visualized with high-frame-rate blood speckle tracking. In the healthy heart (**left**), vortices are compact and apically centered, indicating efficient filling, whereas in congenital heart disease (**right**), they appear enlarged or displaced due to altered ventricular geometry and loading. LA = left atrium; LV = left ventricle; MV = mitral valve; RA = right atrium; RV = right ventricle. Adapted from Marchese et al. [110].

Henry et al. [10] applied BST in children with bicuspid aortic valve and observed eccentric, disorganized flow with increased energy loss and vorticity despite the absence of stenosis or dilation. These changes correlated with anatomical and diastolic indices, suggesting that BST can reveal early hemodynamic alterations. Marchese et al. [110] confirmed the feasibility of vortex imaging with HFR speckle tracking in both healthy and congenital heart disease cohorts, showing that vortex location varied according to ventricular morphology under pressure or volume overload. As illustrated in Figure 5, vortices in healthy hearts are compact and apically positioned, whereas in congenital heart disease, they appear displaced or enlarged, reflecting altered ventricular geometry and loading conditions.

BST has also been used to evaluate right-sided and pulmonary flow [111,112]. In children with pulmonary arterial hypertension, BST revealed abnormal diastolic vortices in the main pulmonary artery and increased energy loss and vector complexity, changes that were absent in controls [111]. Mawad et al. [112] reported similar findings in patients with dilated right ventricles, identifying inefficiencies often missed by conventional imaging.

In adults, Daae et al. [113] employed ECG-gated multi-beat BST to visualize short-lived events such as isovolumic flow initiation and vortex evolution during diastole. Patterns were synchronized with atrioventricular-plane motion and correlated with ventricular geometry, supporting the role of vortices in efficient filling and ejection. L’Hoyes et al. [114] described the potential use of BST for a better positioning of intracardiac circulatory support devices.

Voorneveld et al. [115] validated high-frame-rate echoPIV in a phantom study, showing accurate quantification of high-velocity diastolic flow patterns, including the transmitral jet and vortical structures. The technique was later translated clinically by the same group in a patient with dilated cardiomyopathy [116], capturing peak transmitral velocities and complex intraventricular vortices migrating apically during diastolic filling. Building on this work, they investigated patients with heart failure and established the optimal imaging conditions for reliable quantification of transmitral and left ventricular outflow patterns [117]. Toulemonde et al. [118] demonstrated the feasibility of contrast-enhanced HFR echocardiography in humans, using microbubble tracking to visualize intracardiac flow at high temporal resolution. The group [119,120] extended their method to patients with cardiomyopathy and conduction abnormalities, confirming strong agreement with 4D flow MRI in assessing velocity fields, energy loss, and hemodynamic forces. Both studies revealed reduced vortex strength and altered flow organization in dilated ventricles compared with normal hearts, underscoring the potential of HFR echoPIV for detecting pathological flow remodeling in systolic dysfunction.

In a preclinical porcine model, Wahyulaksana et al. [121] demonstrated that high-frame-rate contrast-enhanced echocardiography can detect myocardial perfusion deficits and reactive hyperemia, supporting the feasibility of assessing coronary microcirculatory flow beyond conventional contrast imaging.

Clinically, high-frame-rate flow imaging allows characterization of vortex formation, flow propagation, intraventricular pressure gradients, and energy loss, providing insight into ventricular filling, energetic efficiency, and abnormal flow patterns in conditions such as valvular disease, cardiomyopathies, and congenital heart disease. However, most clinical evidence currently derives from pediatric or congenital populations, and studies in adult patients remain limited.

## 6. Discussion

HFR echocardiography opens a new frontier in functional assessment of the heart and vessels, but technical and clinical challenges must be resolved before it can be fully translated into routine practice.

### 6.1. Clinical Priorities

Although HFR echocardiography has shown promising clinical added value across several applications, its implementation remains limited and not yet standardized. Acquisition protocols, post-processing methods, and vendor platforms differ widely, which limits reproducibility and comparability across centers [122,123]. In addition, most HFR studies have been performed using research scanners or modified clinical ultrasound systems (see reported setups in the Supplementary Tables), which have further limited their integration into routine clinical workflows [124]. Therefore, data analysis still involves some manual processing steps, as analysis workflows continue to evolve, and requires training and experience, although it does not appear substantially more operator-dependent than other advanced echocardiographic techniques.

Furthermore, most HFR studies have been conducted in single research centers and in relatively small, highly selected cohorts, often excluding patients with arrhythmias or poor image quality [45,49,50,51,112,113]. This selection bias limits the generalizability of the findings and does not fully reflect real-world clinical populations. Accordingly, large multicenter trials validating HFR-derived biomarkers against invasive or established imaging standards remain scarce [44,47,50,51,125].

Further validation of the clinical added value of HFR imaging is required, but it depends on its broader availability on clinical ultrasound systems. Although some vendors of medical ultrasound equipment have begun enabling HFR imaging capabilities on their platforms, its use in routine clinical practice remains impractical. This currently restricts the ability to determine in which clinical scenarios HFR imaging may complement or potentially substitute established techniques such as global longitudinal strain, Doppler echocardiography, or cardiovascular magnetic resonance. Consequently, it remains uncertain for which specific cardiac conditions HFR imaging will provide the greatest clinical benefit. Nevertheless, the currently available literature—as summarized in this review paper—clearly demonstrated the clinical potential of HFR imaging.

### 6.2. Technical Limitations

High-frame-rate acquisition improves temporal resolution and enables capture of brief mechanical and hemodynamic events, but often compromises spatial detail [126]. This trade-off is especially pronounced in patients with poor acoustic windows, where measurement reliability remains limited despite higher frame rates [31,40,126].

Current implementations utilize predominantly two-dimensional echocardiography, limiting the ability to represent the heart’s integrated longitudinal, radial, and circumferential motion. This restriction may introduce systematic errors in estimating strain, stiffness, and flow [43,44]. Although three-dimensional approaches have been proposed [99,123,125,127,128,129,130,131], current systems often rely on ECG gating or multi-beat reconstruction to achieve high frame rate, which reduces feasibility in patients with irregular cardiac rhythms.

Beyond image quality and dimensionality, artifacts remain a major obstacle. Clutter, noise, and beamforming inaccuracies compromise deformation and flow measurements, and while filtering and motion-compensation techniques offer partial improvement [40,42], they do not completely eliminate these errors in clinical practice.

In deformation imaging, advances in temporal resolution have not resolved several inherent limitations that neither arise from nor can be addressed by high-frame-rate imaging. However, these advances have enabled electromechanical wave imaging, which remains largely limited to spontaneous arrhythmic events that provide suitable activation patterns [125,132].

In mechanical wave imaging, restricted spatial and temporal resolution that remains suboptimal despite high-frame-rate acquisition may affect the accuracy of wave speed estimates. Moreover, interpreting mechanical wave propagation speed in relation to pathology is challenging, since wave velocity is influenced not only by the type of mechanical wave but also by loading conditions, myocardial contractility, and intrinsic material properties such as fiber orientation and nonlinear tissue behavior [58,62,79].

In flow imaging, accuracy decreases at greater depths [102], and derived parameters such as vorticity and energy loss remain highly sensitive to post-processing choices, limiting reproducibility across studies [111].

### 6.3. Technical Priorities

In deformation imaging, future work should focus on improving spatial resolution and motion-tracking robustness to better capture regional myocardial function. HFR-STE may overcome the temporal limitations of conventional STE, enhancing sensitivity to subtle or localized dysfunction [32,40,126].

Learning-based approaches can reduce operator dependency by estimating local activation times from HFR strain-rate data and reconstructing the spatiotemporal propagation of myocardial electromechanical activation, with performance comparable to established EWI methods [46,132]. Artificial intelligence also improves segmentation and vector field reconstruction under suboptimal imaging conditions [133]. Integrating EWI-derived metrics with ECG and conventional echocardiography may improve patient selection and guidance for CRT [134]. At a broader level, recent work on AI-driven cognitive ultrasound, in which transmit–receive strategies are adaptively selected using active inference and deep generative modeling, highlights the potential of intelligent systems to personalize data acquisition and improve the efficiency and robustness of echocardiographic imaging [135].

In mechanical wave propagation imaging, advances such as compact anisotropy-sensitive probes, refined excitation strategies, and comprehensive waveform analysis may improve accessibility and diagnostic accuracy [56,73,131,136]. Novel probe designs, including the anisotropy-sensitive transducer developed by Pedreira et al. [137], enable directional stiffness estimates with reduced complexity. Technical innovations such as sliding-window coherent compounding may enhance temporal resolution, though the associated trade-off with image quality must be balanced to ensure clinical feasibility [18]. Beyond direct stiffness estimation, parameters derived from mechanical wave imaging can be combined with deformation and geometric information to compute secondary biomechanical indices, such as regional myocardial work, providing additional insight into contractile function and therapy monitoring [67].

Flow imaging is progressing toward real-time applications, supported by GPU-based reconstruction, adaptive beamforming, and hybrid vector Doppler systems [124,138,139]. Rossi et al. [127] and Guidi et al. [123] developed three-dimensional and real-time flow mapping systems using frequency-domain processing and custom beamforming. Extending this, four-dimensional vector flow imaging was applied in animal models to assess intraventricular flow dynamics and coronary vasculature [140].

Quantitative performance has been validated in phantom and in vivo studies, demonstrating accurate flow estimation under controlled conditions [113,141], while adaptive and nonlinear beamforming promise improved fidelity in deep or challenging regions. In parallel, HFR plane-wave Doppler imaging with adaptive spatiotemporal clutter filtering was used to implement coronary HFR Doppler angiography, permitting visualization of intramyocardial vessels and estimation of coronary flow reserve in a swine model [142].

Future developments in high-frame-rate imaging include ultrasound localization microscopy, which enables super-resolution mapping of myocardial microvascular flow by localizing microbubbles beyond the diffraction limit through repeated HFR acquisitions [141]. Although human experience remains limited, recent transthoracic studies demonstrate the feasibility of reconstructing myocardial microvasculature and flow within short breath holds using motion-compensated HFR imaging, revealing microvascular alterations not detected by conventional angiographic techniques [143]. These early results suggest potential applications of ULM for noninvasive myocardial microvascular assessment.

## 7. Conclusions

HFR ultrasound is an advanced imaging technique that enables the visualization of rapid cardiac events by acquiring up to several thousand frames per second. This improvement in temporal resolution has expanded the scope of echocardiography beyond simple structural and functional assessment, supporting novel applications in deformation imaging, mechanical wave propagation imaging, and flow analysis. Techniques such as EWI allow noninvasive mapping of myocardial activation for arrhythmia characterization and therapy guidance. Mechanical wave propagation imaging provides information on myocardial properties that could not have been obtained so far. HFR flow imaging provides a quantitative evaluation of fast intracardiac hemodynamics, particularly valuable in congenital heart disease and pediatric populations. These HFR ultrasound-based developments mark a shift toward more dynamic and functional cardiac evaluation. As technical challenges continue to be addressed and clinical validation expands, HFR ultrasound is positioned to play a growing role in cardiovascular diagnosis and decision-making.

## Data Availability

No new data were created or analyzed in this study.

## Supplementary Materials

The following supporting information can be downloaded at: https://www.mdpi.com/article/10.3390/jcm15062460/s1, Table S1. An overview of reviewed clinical papers of motion and deformation imaging; Table S2. An overview of reviewed clinical papers of mechanical wave propagation imaging; Table S3. An overview of reviewed clinical papers of flow imaging.

## Abbreviations

The following abbreviations are used in this manuscript:

HFR	High Frame Rate
ASE	American Society of Echocardiography
ECG	Electrocardiogram
TDI	Tissue Doppler Imaging
STE	Speckle-Tracking Echocardiography
fps	Frames per Second
EWI	Electromechanical Wave Imaging
ECLM	Electromechanical Cycle-Length Mapping
SW	Shear Wave
iVP	Intrinsic Velocity Propagation
MS	Myocardial Stiffness
SWE	Shear Wave Elastography
ARF	Acoustic Radiation Force
CMR	Cardiovascular Magnetic Resonance
SWS	Shear Wave Speed
HCM	Hypertrophic Cardiomyopathy
CA	Cardiac Amyloidosis
CRT	Cardiac Resynchronization Therapy
LBBB	Left Bundle Branch Block
RV	Right Ventricle/Right Ventricular
LV	Left Ventricle/Left Ventricular
BST	Blood Speckle Tracking
echoPIV	Echocardiographic Particle Image Velocimetry
IVPD	Intraventricular Pressure Difference
ULM	Ultrasound Localization Microscopy
