# Supplementary material for: High-Frame-Rate Echocardiography: A New Frontier in Noninvasive Functional Assessment
Source: J Clin Med. 2026 Mar 23;15(6):2460. doi: 10.3390/jcm15062460 (PMC13026948; doi:10.3390/jcm15062460)
Supplement: Supplementary file 1 [file jcm-15-02460-s001.zip › jcm-4184642-supplementary.pdf]

Journal of
Clinical Medicine

Supplementary Materials:

Note: A dash (–) in the tables indicates that the corresponding information was not explicitly reported in the referenced study.

Table S1. An overview of reviewed clinical papers of motion and deformation imaging

Ref	Reported Setup	Frame Rate (fps)	Application	Study Subject
**Strain and Strain-Rate Imaging**				
Joos et al. [37]	GE Vivid q and Verasonics	500	Quantify the 2-D motion and tissue velocities of the left ventricle	Phantom, 10 healthy volunteers
Slørdahl et al. [38]	GE Vingmed System Five ultrasound scanner; phased-array cardiac probe	323	Feasibility of HFR strain-rate imaging to resolve rapid myocardial deformation patterns and timing of systolic and diastolic events in the interventricular septum	9 healthy volunteers
Orlowska et al. [11]	ULA-OP 256 research scanner; PA230 phased-array probe	833	Assessment of strain and strain-rate response during pharmacological stress at increasing workloads	25 healthy volunteers
Papangelopoulou et al. [39]	HD-PULSE experimental scanner; 3.5 MHz phased-array probe	915 ± 85	Validation of diastolic strain-rate biomarkers (SR_IVR and SR_E) using high-frame-rate speckle tracking against tissue Doppler reference	28 patients with cardiac amyloidosis, 10 with hypertrophic cardiomyopathy, 16 healthy volunteers
Porée et al. [40]	Verasonics V-1-128 research scanner; ATL P4-2 phased-array probe	500	Development and validation of a dual tissue-Doppler–optical-flow framework for accurate 2-D myocardial velocity estimation at high frame rate	Phantom,<br>5 healthy volunteers
Schleifer et al. [41]	Verasonics Vantage research system; ATL P4-2 phased-array probe	300	Feasibility of high-frame-rate myocardial elastography using maximum principal strain for early myocardial infarction detection in emergency-department patients	10 patients with chest pain; 7 analyzed
El Harake et al. [42]	Verasonics Vantage 256 research system; ATL P4-2 phased-array probe	300	Preliminary feasibility of stress myocardial elastography for detection of coronary artery disease validated against nuclear perfusion imaging	2 canines with induced ischemia; 8 patients undergoing pharmacological stress testing for suspected CAD
**Electromechanical Activation Imaging (EWI, ECLM, CFWI)**				
Provost et al. [43]	Verasonics Vantage with a 2.5-MHz phased-array probe	1000-2000	Evaluate the effectiveness of EWI in mapping the electromechanical activity of the atria during focal and reentrant arrhythmias	8 patients (1 with premature ventricular complex, 5 with atrial flutter, 1 with focal atrial tachycardia, 1 with

				atrial fibrillation) and 1 healthy control
Bessière et al. [44]	Verasonics Vantage with L15-Xtech Vermon probe	2500 and 4000	Experimental validation of high-frame-rate EWI for differentiating endocardial vs epicardial ventricular activation in isolated working-mode hearts	4 isolated swine hearts
Tonko et al. [45]	Verasonics Vantage with a 2.5 MHz phased-array	2000	Clinical validation of high-frame-rate electromechanical wave imaging for localization of ventricular arrhythmia origins against invasive contact mapping	20 patients with ventricular arrhythmias
Safarzadeh et al. [46]	GE Vivid E95 with 3.5 MHz phased-array probe	824 ± 91	Development of a patient-specific high-frame-rate speckle-tracking method to automatically determine local activation times from strain-rate curves	20 healthy volunteers, 20 heart failure patients treated with CRT
Tourni et al. [47]	Vantage 256 with ATL P4–2 phased array probe	2000	Evaluate the feasibility and clinical utility of using EWI to assess and characterise MV disease in pediatric patients	1 open-chest healthy canine , 5 pediatric subjects with mitral valve prolapse and 5 healthy controls
Melki et al. [48]	Verasonics Vantage, ATL P4–2 phased array probe	2000	Develop and evaluate a novel metric that quantifies the dispersion of electromechanical activation in the ventricles	4 patients with His-bundle pacing, 15 with biventricular CRT, 10 with chronic RV pacing, 10 with LBBB without pacing, and 4 healthy controls
Tourni et al. [49]	Verasonics Vantage 256 with ATL P4–2 phased array probe	2000	Evaluate the feasibility of using ECLM feasibility to map and quantify atrial arrhythmic activation rates, assess the effectiveness of DCCV in converting AF to normal sinus rhythm, and predict short-term and long-term outcomes following DCCV treatment	30 patients with atrial fibrillation undergoing direct-current cardioversion and 15 healthy volunteers
Tourni et al. [50]	Verasonics Vantage 256 with P4–2 phased array	2000	Develop and evaluate the feasibility of ECLM to map and quantify AF electromechanical CL activity and inform on long-term ablation procedure response	16 patients with atrial fibrillation, 6 with atrial flutter
Tourni et al. [51]	Verasonics Vantage 256 with P4–2 phased array	2000	Evaluate the feasibility and effectiveness of using 3D ECLM to map atrial arrhythmias and assess pulmonary vein isolation ablation outcomes	15 patients with atrial fibrillation

Kvale et al. [68]	GE Vivid E95, a 2.8 MHz phased array	1000-1200	Develop and evaluate a non-invasive method for mapping the regional mechanical activation of the LV myocardium using HFR ultrasound imaging	open-chest dogs with: sinus rhythm (5), RV free wall pacing (4), LV free wall pacing (3), BiV (2) and septal (1) pacing
Grondin et al. [125]	4 synchronized Verasonic Vantages with a 3.6 MHz, 32x32 elements matrix array Vermon probe	840 (Volumes-s)	Demonstrate the feasibility of using high volume-rate echocardiography to simultaneously map both electromechanical activation and cardiac strain of the entire heart in a single heartbeat	20 healthy volunteers, 16 analyzed
Melki et al. [132]	Verasonics Vantage 256 with ATL P4–2 Philips phased array probe	2000	Combination of EWI with machine learning for automated generation of electromechanical isochrone maps to reduce operator dependency	24 patients with Wolff-Parkinson-White, 5 healthy volunteers, 6 different LV-paced canine

Table S2. An overview of reviewed clinical papers of mechanical wave propagation imaging.

Ref	Reported Setup	Frame Rate (fps)	Application	Study Subject
**Shear Wave Elastography**				
Bezy et al. [52]	GE Vivid E95 with HD-PULSE (research scanner)	1,304 ± 115	Myocardial stiffness assessment during ischemia–reperfusion injury	15 pigs (13 completed)
Strachinaru et al. [53]	Philips iE33 with S5-1 probe and Verasonics Vantage with L7-4	~510 Hz (TDI)	Detection of local myocardial stiffness variations	Phantom, 10 healthy, 10 hypertrophic cardiomyopathy, 10 hypertrophic cardiomyopathy post-septal reduction
Villemain et al. [54]	Aixplorer (SuperSonic Imagine) with 2.75 MHz phased-array probe	-	Assessment of diastolic myocardial stiffness in pediatric hypertrophic cardiomyopathy	28 patients with hypertrophic cardiomyopathy, 28 healthy controls
Song et al. [55]	Verasonics Vantage with Philips P7-4 (pediatric) and P4-2 (adult) probes	-	Establishment of pediatric myocardial stiffness reference data	20 healthy children
Caenen et al. [56]	Verasonics Vantage 256 with P4-2 and P4-2V probes	6200	Transthoracic shear wave elastography for dynamic stiffness evaluation	13 pigs (Yorkshire–Landrace), 7 successful acquisitions
Malik et al. [59]	Verasonics Vantage 256 with GE 6S-D phased-array probe	-	Assessment of myocardial stiffness throughout the cardiac cycle in healthy and hypertrophic cardiomyopathy	20 healthy volunteers, 20 patients with hypertrophic cardiomyopathy
Vos et al. [60]	SonixTOUCH (Ultrasonix) with SA4-2 phased-array probe	-	Noninvasive myocardial stiffness assessment in a porcine model	22 healthy Göttingen minipigs
Brekke et al. [61]	GE Vivid E9 ultrasound system; M5S-D phased-array probe	1200	Ultra-high-frame-rate TDI for quantifying myocardial velocities and timing of electromechanical events, including pre-ejection dynamics and mechanical wave propagation associated with aortic valve closure	10 healthy volunteers; 1 patient with atrial fibrillation
Coudae et al. [63]	Supersonic Imagine prototype with 128-element linear probe (8 MHz)	12000	Real-time mapping of myocardial stiffness and anisotropy throughout the cardiac cycle	10 open-chest sheep
Pernot et al. [64]	Supersonic Imagine Aixplorer with 128-element linear probe (Vermon)	12000	Quantitative mapping of myocardial stiffness and contractility	6 Langendorff-perfused isolated rat hearts
Pernot et al. [65]	ultrafast ultrasonic scanner (Aixplorer, SuperSonic	10000	Quantification of passive diastolic myocardial stiffness in ischemic cardiomyopathy (comparison	10 sheep: 5 with 15 min left anterior descending coronary occlusion + 40 min reperfusion

	Imagine) with linear array (SuperSonic Imagine) probe		between stunned and infarcted myocardium)	(stunned group); 5 with 120 min occlusion + 40 min reperfusion (infarcted group)
Villemain et al. [66]	Aixplorer (SuperSonic Imagine) with 2.75 MHz phased-array (SuperSonic Imagine) probe	-	Quantification of diastolic myocardial stiffness	60 healthy adults, 20 patients with hypertrophic cardiomyopathy and heart failure with preserved ejection fraction
Venet et al. [67]	GE Vivid E95 and Verasonics Vantage; GE 6S-D or M5Sc-D prob	-	Noninvasive estimation of myocardial work using stiffness and strain	20 healthy children, 20 with hypertrophic cardiomyopathy, 5 with aortic valve stenosis
Meyer et al. [69]	GE Vivid E9 and 2 MHz phased-array probe (GAMPT)	100 (prior to temporal up-sampling)	Quantification of diastolic myocardial stiffness in heart failure with preserved ejection fraction and amyloidosis	54 healthy controls, 10 with mild left ventricular hypertrophy, 45 with wild-type transthyretin amyloidosis (20 treated)
Kanai et al. [71]	Custom ultrasound phased-tracking system	450	In vivo measurement of spontaneous pulsive wave propagation after aortic valve closure to estimate myocardial viscoelasticity (first noninvasive viscoelasticity estimation in humans)	5 healthy volunteers
Santos et al. [72]	GE Vivid E9 and HD-PULSE; M5S and P2-5AC probes	-	Myocardial stiffness assessment at end-diastole and end-systole	30 healthy volunteers, 2 patients (1 with cardiac amyloidosis, 1 undergoing stress echocardiography
Malik et al. [73]	GE Vivid E95 and Verasonics Vantage with GE M5S-D or 6S-D probe	-	Pediatric myocardial stiffness quantification	60 healthy subjects (15 neonates, 45 aged 1 month–45 years)
Youssef et al. [74]	GE Vivid E95 with M5Sc-D probe	1295 ± 164	Assessment of myocardial stiffness and diastolic function across age groups	106 healthy children, 62 healthy adults
Strachinaru et al. [75]	Philips iE33 with S5-1 probe	500-590	Assessment of myocardial stiffness in healthy individuals and patients with hypertrophic cardiomyopathy	45 healthy individuals, 43 with hypertrophic cardiomyopathy
Petrescu et al. [76]	HD-PULSE (research scanner) with Medison P2-5AC phased-array probe	1,150 ± 245	Myocardial stiffness assessment using natural shear waves	46 healthy volunteers (3 age groups), 17 patients with cardiac amyloidosis
Cafezeiro et al. [77]	Canon APLIO i800 with 3.5 MHz convex probe	-	Comparison of myocardial stiffness in cardiac amyloidosis and Fabry disease	20 participants with cardiac amyloidosis, 20 with Fabry disease, 20 healthy controls
Wouters et al. [78]	GE Vivid E95 with M5Sc-D probe	1032 ± 125	Assessment of myocardial stiffness in valve disease and after replacement	17 patients with aortic valve stenosis, 13 with transcatheter valve replacement, 10 with

				surgical valve replacement, 18 healthy individuals
Espeland et al. [79]	GE Vivid E95 with M5Sc-D probe	858-1403	Assessment of myocardial stiffness in healthy individuals and patients with aortic valve stenosis	63 healthy participants, 13 patients with severe aortic valve stenosis
Wouters et al. [80]	Programmable experimental scanner; probe not specified	-	Detection of septal scarring in patients with left bundle branch block undergoing cardiac resynchronization therapy	29 participants with left bundle branch block without scar, 10 with septal scar, 11 healthy controls
Petrescu et al. [81]	Custom HD-PULSE scanner with Medison P2-5AC probe	1135 ± 270	Assessment of diastolic myocardial stiffness in heart transplant recipients	52 heart transplant recipients (46 analyzed)
Cvijic et al. [82]	HD-PULSE system with P2-5AC probe	1266 ± 317	Assessment of myocardial stiffness in hypertensive heart disease	33 patients with arterial hypertension (13 with concentric remodeling, 20 with concentric hypertrophy), 26 healthy controls
Werner et al. [83]	HD-PULSE experimental scanner with Samsung P2-5AC probe	1050 ± 220	Noninvasive estimation of left ventricular filling pressures	85 patients undergoing catheterization; 16 with decompensated heart failure for follow-up
Salaets et al. [85]	Verasonics Vantage system	-	Assessment of diastolic myocardial stiffness in children with Fontan circulation	24 children with Fontan circulation (single-ventricle physiology), 33 age-matched healthy controls
Youssef et al. [86]	Programmable experimental scanner	-	Monitoring myocardial stiffness recovery in children with multisystem inflammatory syndrome after COVID-19	7 children with multisystem inflammatory syndrome, 30 age-matched healthy controls
Venet et al. [91]	GE Vivid E9 system and Verasonics Vantage 256 with Vrasonics L22-14vX probe and GE 6S-D phased array probe	20000 (Virtual); 2800 (Tissue Doppler)	Assessment of myocardial stiffness in right ventricular pressure overload	19 rats (6 pulmonary artery banding, 7 Sugen-hypoxia, 6 sham) and 14 children (7 with right ventricular pressure overload, 7 healthy)
Alencar et al. [92]	Canon APLIO i800 ultrasound system with 3.5 MHz convex probe	-	Assessment of right ventricular stiffness in transthyretin-related cardiac amyloidosis	20 adults with cardiac transthyretin amyloidosis, 20 with non-cardiac transthyretin amyloidosis, 20 healthy controls
**Stretch Wave Imaging**				
Pislaru et al. [57]	GE Vivid E9 or Vivid Q and Philips iE33	350-460	Assessment of myocardial stiffness in valvular heart disease	20 patients with aortic stenosis, 20 with mitral regurgitation, 20 healthy controls
Pislaru et al. [58]	GE Vivid E9 ultrasound system	350-450	Measurement of late-diastolic myocardial intrinsic velocity wave	16 pigs; 10 healthy volunteers

			propagation to assess myocardial stiffness and preload dependence using HFR TDI	
Kvale et al. [68]	Modified GE Vivid E95 ultrasound system; phased-array probe (2.8 MHz center frequency)	1000-1200	Noninvasive mapping of regional left ventricular mechanical activation and activation sequence using clutter filter wave imaging during sinus rhythm and pacing	5 open-chest canine models
Støylen et al. [93]	GE Vingmed System Five ultrasound scanner; 2.5-MHz phased-array transducer	70-100	Quantification of diastolic myocardial deformation and intrinsic velocity propagation using strain-rate imaging to characterize normal and impaired diastolic function	28 healthy subjects; 26 patients with hypertension and preserved systolic function
Pislaru et al. [94]	GE Vivid E9/E95	250-465	Assessment of myocardial stiffness in cardiac amyloidosis	67 patients with amyloidosis (48 cardiac, 19 non-cardiac) and 40 healthy controls
Pislaru et al. [95]	GE E9 or E95 clinical scanner	250-465	Intrinsic cardiac elastography for noninvasive estimation of myocardial stiffness using naturally occurring diastolic stretch waves; validation in amyloidosis with prognostic correlation	56 patients with systemic amyloidosis (38 with cardiac involvement, 18 without) and 38 controls
Benz et al. [96]	High–frame-rate echocardiography; specific machine/probe not specified	>250	Myocardial stiffness in cardiac amyloidosis, hypertrophic cardiomyopathy, and healthy controls	28 patients with cardiac amyloidosis, 10 with hypertrophic cardiomyopathy, 16 healthy volunteers
Strachinaru et al. [97]	Philips iE33 ultrasound system; S5-1 phased-array transducer	>500	Quantification of myocardial intrinsic velocity propagation following atrial contraction using HFR color tissue Doppler imaging to characterize septal mechanical behavior in normal and hypertrophic myocardium	42 healthy volunteers; 33 patients with hypertrophic cardiomyopathy
Halvorsrod et al. [98]	GE Vivid E95 ultrasound system; 4Vc-D transducer	750	Estimation of atrial-kick–induced myocardial mechanical wave velocities using three-dimensional high-frame-rate echocardiography to characterize myocardial tissue properties in acute myocardial infarction	20 patients with ST-elevation myocardial infarction; 20 healthy controls

Salles et al. [99]	Modified GE Vivid E95 ultrasound system; 4V matrix array transducer	820	Three-dimensional mapping of atrial-kick–induced myocardial mechanical wave velocities using clutter filter wave imaging to characterize left ventricular mechanical properties	5 healthy volunteers; 10 patients with aortic stenosis
Andresen et al. [100]	GE Vivid E95	1180 (1134–1199)	Feasibility of intrinsic mechanical wave velocity measurement (AK, MVC, AVC) using CFWI to detect regional myocardial dysfunction after acute coronary syndrome	60 patients with acute coronary syndrome after revascularization

Table S3. An overview of reviewed clinical papers of flow imaging.

Ref	Reported Setup	Frame Rate (fps)	Application	Study Subject
**Blood Speckle Tracking and echoPIV**				
Nyrnes et al. [102]	GE Vivid E9 with GE linear (9L) and phased-array (6S, 12S) probes	5000-7000	Pediatric and fetal cardiology: assessment of septal defects, valvular stenosis, pulmonary hypertension, and cardiomyopathies	4 fetuses participants , 51 healthy controls, 47 cardiac patients
Fadnes et al. [107]	SonixMDP system with 4–9 MHz linear transducer	Flow: 107; B-mode: 54	Assessment of ventricular and atrial septal defects in congenital heart disease	2 neonates: one with ventricular and atrial septal defects and additional anomalies, one with isolated atrial septal defect
Cantinotti et al. [108]	GE Vivid TM E95 with 6S and 12S phased-array probes	400–500	Evaluation of congenital heart disease in neonates (including transposition of the great arteries, septal defects, atrioventricular septal defect, aortic and pulmonary stenosis, tetralogy of Fallot, total anomalous pulmonary venous return, and aortic coarctation)	8 neonates with various congenital heart disease diagnoses
Sørensen et al. [8]	GE Vivid E9/E90/E95 with 6S, 9L, and 12S probes	33-53	Estimation of early diastolic intraventricular pressure difference in children	169 participants: 138 controls, 10 with dilated cardiomyopathy, 21 with hypertrophic cardiomyopathy
Sørensen et al. [109]	GE Vivid E90 with 6S phased-array probe	-	Longitudinal assessment of left ventricular diastolic flow maturation in healthy neonates	36 term newborns with 176 echocardiographic exams across five timepoints
Henry et al. [10]	GE Vivid E9 with 6S/12S phased-array probes	-	Assessment of flow complexity in bicuspid aortic valve	38 children: 14 with bicuspid aortic valve, 24 healthy controls
Marchese et al. [110]	GE Vivid E95 with 12S and 6S probes	380	Assessment of left ventricular vortex characteristics	118 healthy children, 43 with congenital heart disease
Mawad et al. [111]	GE Vivid E9/E95 with 6s and 12s phased array probes	-	Assessment of pulmonary artery and right ventricular flow in pulmonary hypertension	18 children with pulmonary arterial hypertension, 18 healthy controls
Mawad et al. [112]	GE Vivid E9 with 6S and 12S phased-array probes	-	Right ventricular energy loss and vorticity mapping	21 children with repaired tetralogy of Fallot, 11 with atrial septal defect, 25 healthy controls
Daae et al. [113]	GE E95 with 4V-D and M5S-c probe	>3500	Assessment of left ventricular vortex and flow dynamics	21 healthy adults
L'Hoyes et al. [114]	GE Vingmed E95 System; 6VT-D TEE probe and 6S-D probe	400-500	Angle-independent visualization of intracardiac and great-vessel blood flow using blood speckle	Critically ill patient undergoing veno-arterial extracorporeal

			imaging for device positioning and flow assessment during percutaneous mechanical circulatory support	membrane oxygenation with Impella CP support
Voorneveld et al. [116]	Philips EPIQ 7 (X5-1 probe), Verasonics Vantage 256 (P4-1 probe), with SonoVue contrast	1225	Assessment of left ventricular diastolic flow using high-frame-rate contrast-enhanced echo particle image velocimetry (echo-PIV)	1 patient with dilated cardiomyopathy and dual-chamber implantable cardioverter-defibrillator
Voorneveld et al. [117]	Verasonics Vantage 256; ATL P4-1 phased-array probe; SonoVue contrast	1225	Optimization of contrast infusion rate and transmit voltage for HFR echoPIV and validation against PW Doppler in LV inflow/outflow	20 patients with heart failure
Toulemonde et al. [118]	Verasonics Vantage system with ATL P4-1 phased-array probe	5500	High-frame-rate contrast echocardiography for simultaneous chamber-flow and myocardial-perfusion imaging	Healthy adult volunteer
Han et al. [119]	Verasonics Vantage 256; ATL P4-1 phased-array probe; SonoVue contrast	Acquisition ≈ 3553-6012 (raw PRF); processed ≈ 122.5	Validation of HFR echoPIV against 4D flow MRI for LV velocity, kinetic energy, and energy loss quantification	26 patients referred for cardiac MRI
Han et al. [120]	GE Vivid E95; SonoVue contrast	100	Comparison of hemodynamic forces and vortex circulation between HFR echoPIV and 4D flow MRI	26 patients with normal and dilated LV function
Wahyulaksana et al. [121]	Verasonics Vantage 256 research ultrasound system; ATL Philips P7-4 phased-array probe (center frequency 5.2 MHz)	500	HFR contrast-enhanced ultrasound combined with higher-order singular value decomposition to visualize myocardial perfusion deficits and differentiate fast and slow coronary microcirculatory flow during acute coronary occlusion	Porcine model with transient left anterior descending coronary artery occlusion and release
**Ultrasound Localization Microscopy**				
Yan et al. [143]	Verasonics Vantage 256 with GE M5Sc-D probe and SonoVue contrast agent	305	Imaging of myocardial microvasculature using ultrasound localization microscopy (ULM)	2 patients with hypertrophic cardiomyopathy, 1 with dilated left ventricle due to ectopy, 1 with idiopathic ventricular fibrillation
